# Association Analysis of the HNF4A Common Genetic Variants with Type 2 Diabetes Mellitus Risk

**DOI:** 10.22088/IJMCM.BUMS.8.2.56

**Published:** 2019-08-28

**Authors:** Seyedeh Mina Azizi, Negar Sarhangi, Mahdi Afshari, Davood Abbasi, Hamid Reza Aghaei Meybodi, Mandana Hasanzad

**Affiliations:** 1 *Medical Genomics Research Center, Tehran Medical Sciences, Islamic Azad University, Tehran, Iran.*; 2 *Personalized Medicine Research Center, Endocrinology and Metabolism Clinical Sciences Institute, Tehran University of Medical Sciences, Tehran, Iran.*; 3 *Department of Community Medicine, Zabol University of Medical Sciences, Zabol, Iran.*; 4 *Iranian Diabetes Society, Eslamshahr Branch, Iran.*; 5 *Endocrinology and Metabolism Research Center, Endocrinology and Metabolism Clinical Sciences Institute, Tehran University of Medical Sciences, Tehran, Iran.*

**Keywords:** T2DM, T2DM complication, HNF4A, gene, sequencing

## Abstract

Type 2 diabetes mellitus (T2DM) is a complex disease that involves a wide range of genetic and environmental factors. The *hepatocyte nuclear factor (HNF4A)* carries out hepatic gluconeogenesis regulation and insulin secretion crucially, and the corresponding gene was shown to be linked to T2DM in several studies. The aim of the present study was to evaluate the association between *HNF4A* genetic variants (rs1884613 and rs1884614) and T2DM risk in a group of Iranian patients. This case-control study included 100 patients with T2DM and 100 control subjects. Genotyping of two single nucleotide polymorphisms (SNPs) (rs1884613 and rs1884614) of *HNF4A* was performed using the sequencing method. There was no statistically significant difference for allele and genotype distribution of the *HNF4A* common variants (rs1884613 and rs1884614) between subjects with and without T2DM (P=0.9 and P=0.9, respectively). Regarding diabetic complications, although the presence of mentioned polymorphisms increased the odds of developing ophthalmic complications and reduction of the odds of renal complications among diabetic patients, the mentioned risk was non- significant and cannot be generalized to the whole population.  It seems that rs1884613 and rs1884614 polymorphisms are not associated with T2DM or its renal and ophthalmic complications. To investigate the precise influence of these polymorphisms, prospective cohorts with larger sample sizes are required.

Type 2 diabetes mellitus (T2DM) is a prevalent chronic disorder which results from the genetic and environmental factors. Micro and macrovascular complications may be associated with T2DM (1). It was estimated that in 2015, 415 million people were suffering from type 2 diabetes (T2D) worldwide, which will have risen to 642 million people by 2040 (2).

From pathological point of view, T2DM is characterized by insulin resistance and pancreatic β cell dysfunction (3). Previous similar studies of T2DM in various ethnic populations have demonstrated the association of some genetic variants and β cell dysfunction (4).

The *hepatocyte nuclear factor 4 alpha (HNF4-A)* as a highly conserved transcription factor is expressed in the pancreatic beta cells and many other tissues (5), and plays a pivotal role in the islet beta and liver cells in order to maintain the glucose hemostasis (6). *HNF4-A* extends 29 kb on chromosome 20q13.1-13.2 (7),and includes two distinct promoters, the p1 and p2, with the latter one being located ~ 46 kb downstream of the first one (8-10). *HNF4-A* has been introduced as an important gene in T2DM risk in many studies (11, 12). Also, connection between haplotype of p2 promoter variants of *HNF4-A* and T2DM risk hasbeen recognized in several populations, suggesting that the p2 promoter region may represent a susceptibility locus for T2DM risk (13). The aim of the present study was to assess possible association between *HNF4-A *(rs1884613 and rs1884614) polymorphisms with the risk of T2DM and related complications including retinopathy and nephropathy in a group of Iranian patients for the first time.

**Fig. 1:**
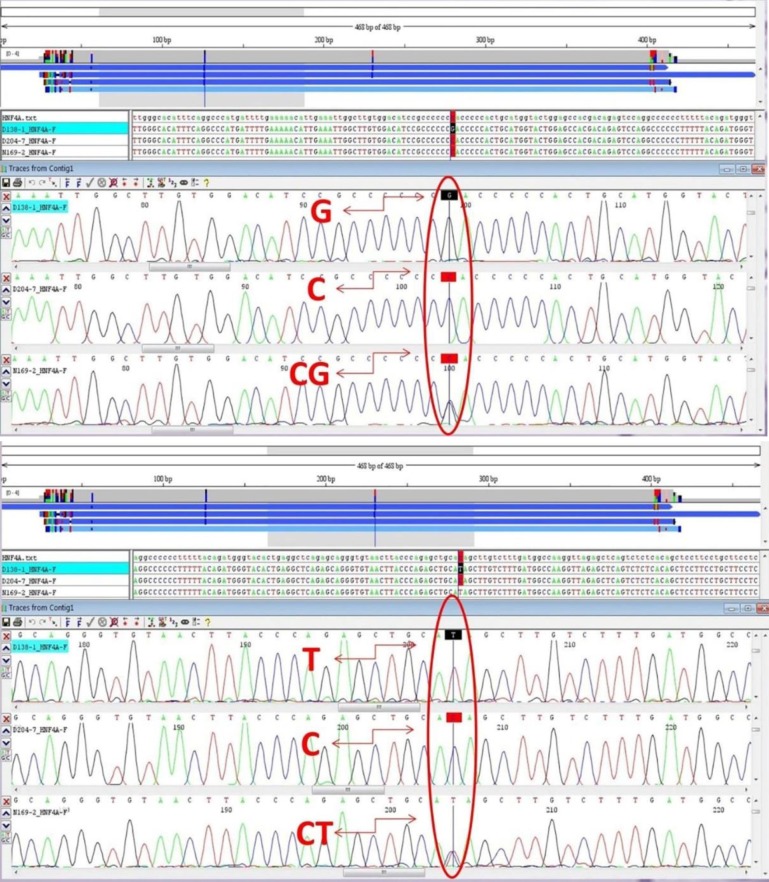
Representative Sanger sequencing results.Upper panel:rs1884613 genotypes: CC, CG and GG;lower panel:rs1884614 genotypes: CC, CT, TT

## Materials and methods

**Patient’s characteristics**

A case-control study including 200 Iranian subjects comprising 100 T2DM patients (cases) and 100 non-diabetic subjects (control) was carried out. Cases were diagnosed strictly according to the American Diabetes Association (ADA) standards criteria (14). Control group included individuals without diabetes that were evaluated according to the components of the diabetes evaluation of ADA guideline (14). Informed written consent was obtained from all subjects before participation and this study was performed in accordance with the

ethical code IR.IAU.TMU.REC.1395.21.

**Genotyping**

Genomic DNA was extracted from samples collected in tubes containing EDTA using the standard protocol (15). For *HNF4A *rs1884613 and rs1884614 genotyping, polymerase chain reaction (PCR) amplification method followed by sequencing was used. Specific primers for the PCR were designed using the primer 3 online primer designer. DNA sequences of primers were 5’-TCTCATAACAGTCAACCAGTTTCTG-3’ and 3’-AGGCAGAGATAGAACGGACAG-5’ for both rs1884613 and rs1884614 from *HNF4A* gene. PCR amplification was carried in 32.8 μl final reaction containing 15 μl dH2O, 15 μl Red Master Mix (Amplicon, UK), 1.5 μl from each of forward and reverse primers (10 pMole/μl) and finally added 1μl DNA template. The cycling conditions were as follows:an initial denaturation step at 95 °C for 5 min, followed by 35 cycles of 1 min at 95°C, 40 s at 60°C and 40 s at 74°C, with a final incubation at 74°C for 5 min. PCR products were separated on a 2% agarose gel, and the quality was confirmed. Sanger sequencing method was used for detection of the *HNF4A* genetic variants (Fig. 1 a and b).

**Statistical analysis**

Frequencies of genotypes /alleles of polymorphisms were compared between patients and control group as well as between patients with and without complications using the Chi- square test. The associations between these genotypes/ alleles and T2DM or its complications were analyzed using logistic regression models. Stata 14 software was used for data analysis. A P-value of less than 0.05 was considered statistically significant.

## Results

200 subjects including 100 T2DM patients and 100 healthy individuals were recruited. Of them, 61 (30.5%) were male. Their mean (SD) age was 48.49 (17.71) years. Patients with T2DM and control subjects had the same gender distribution and were also significantly older than healthy individuals (table 1).

As shown in table 2, the frequencies of CC, CG, and GG genotypes for rs1884613 polymorphism located in the *HNF4A *P2 promoter were 61, 34, and 5% in the T2DM patients and 61, 33, and 6% in control subjects, respectively. That was also the case for CC, CT and TT genotypes of rs1884614 polymorphism.

The frequencies of G allele (rs1884613) as well as T allele (rs1884614) polymorphism among diabetic and non-diabetic subjects were 28.20% vs. 29.03%, respectively. The odds ratio for diabetes mellitus for these two alleles was 0.83 which was not statistically significant (P=0.8) (Table 3).

The distribution of genotypes and allele frequencies of *HNF4 A *common variants (rs1884613 and rs1884614) in the T2DM group did not differ from those in the control group (P=0.9).

According to the results of Tables 4 and 5, diabetic patients with ophthalmic complication did not have a significantly higher frequency of rs1884613 and rs1884614 polymorphisms in comparison with complication-free patients (41.18% vs. 39.02% respectively; P=0.9). In addition, 22.22% and 41.11% of patients with and without renal complications respectively carried rs1884613 and rs1884614 polymorphisms (P=0.5). Among diabetic patients, we also found similar frequencies of these polymorphisms between patients with and without renal and ophthalmic complications.

**Table 1:** Distribution of different factors among subjects with and without T2DM

**Factors**	**Normal subjects** **(N = 100)**	**T2DM** **(N = 100)**	**P-value**
Gender (Female) N (%)	71 (%71)	68 (%61)	0.6
Age (year) (Mean±SD)	34.01±14.23	58.29±12.30	0.0001٭
BMI (Kg/m2) (Mean±SD)	23.73±3.48	28.84±7.91	0.01


**Table 2:** Genotypes distribution of rs1884613 and rs1884614 polymorphisms between two groups, and T2DM risk of HNF4Apolymorphisms

**Genotypes**	**Normal Subjects** **(N = 100)**	**T2DM** **(N = 100)**	**P-value**	**OR**	**95% CI**		**P-value**
**rs1884613**							
CC	61(61%)	61 (61%)	0.99	1	-	-	-
CG	33 (33%)	34 (34%)		1.03	0.56	1.87	0.9
GG	6 (6%)	5 (5%)		0.83	0.24	2.87	0.7
**rs1884614**							
CC	61 (61%)	61 (61%)	0.99	1	-	-	-
CT	33 (33%)	34 (34%)		1.03	0.56	1.87	0.9
TT	6 (6%)	5 (5%)		0.83	0.24	2.87	0.7


**Table 3:** Alleles distribution of rs1884613 and rs1884614 polymorphisms between two groups, and T2DM risk of HNF4Apolymorphism

**Polymorphism**	**Alleles**	**Normal Subjects**	**T2DM**	**P-value**	**OR (P-value)**
rs1884613	C	%77.50	%78	0.9	1
	G	%29.03	%28.20		0.83 (0.8)
rs1884614	C	%77.50	%78	0.9	1
	T	%29.03	%28.20		0.83 (0.8)


**Table 4:** Associations between rs1884613 genotypes and T2DM complications

**Genotypes**	**No complication** **N (%)**	**Ophthalmic complication** **N (%)**	**P-value**	**OR (P-value)**	**95% CI**
CC	50 (60.98)	10 (58.82)	0.9	1	-
CG+ GG	32 (39.02)	7 (41.18)		1.09 (0.9)	0.38-3.17
**Genotypes**	**No complication** **N (%)**	**Renal complication** **N (%)**	**P-value**	**OR (P-value)**	**95% CI**
CC	53 (58.89)	7 (77.78)	0.5		
CG + GG	37 (41.11)	2 (22.22)		0.41 (0.3)	0.08-2.08


**Table 5:** Associations between rs1884614 genotypes and T2DM complications

**Genotypes**	**No complication** **N (%)**	**Ophthalmic complication** **N (%)**	**P-value**	**OR (P-value)**	**95% CI**
CC	50 (60.98)	10 (58.82)	0.9	1	
CG+ GG	32 (39.02)	7 (41.18)		1.09 (0.9)	0.38-3.17
**Genotypes**	**No complication** **N (%)**	**Renal complication** **N (%)**	**P-value**	**OR (P-value)**	**95% CI**
CC	53 (58.89)	7 (77.78)	0.5		
CG + GG	37 (41.11)	2 (22.22)		0.41 (0.3)	0.08-2.08


It should be noted that the above-mentioned risk or protective effects were non- significant and can’t be generalized to the whole population.

## Discussion

We designed this case-control study to search the association of two variants (rs1884613 and rs1884614) of *HNF4A* gene withT2DM risk. In our investigation, we observed no association between *HNF4A* variants rs1884613 and rs1884614 and T2DM risk and also its renal and ophthalmic complications. Despite our negative results, a case-control study by Hansen et al. in the Danish population showed an association between minor T-allele of the rs1884614 with T2DM (16). Love-Gregory et al. in 2004 evaluated the associate on between SNPs, including rsl884613 and rsl884614 in the *HNF4α* gene and T2DM susceptibility, which revealed a close association between these SNPs and T2DM risk in German Jewish individuals (17). On the other hand, two independent studies on Ashkenazi population by Barroso et al. and Neuman et al. demonstrated that rs1884613 SNP of *HNF4A* gene may have an influence on susceptibility to T2DM (18, 19).

In 2005, Winckler et al. provided an updated meta-analysis with patients and control subjects from Sweden, Finland, and Canada, but their results failed to replicate an association between variants of *HNF4A* (rs1884613) and T2DM risk (20).

A significant association between the rs1884614and T2DM susceptibility was not reported by Hara et al. and Tokunaga et al. in two different Japanese populations(13, 21).

In addition, Chen et al. showed that no individual single nucleotide polymorphisms of *HNF4A* including rs1884614 were associated with T2DM risk at either allele or genotype level in the Chinese Han population (22).

Vaxillaire et al. in their study revealed that none of the SNPs (rs1884614) near the beta cell promoter P2 of *HNF-4α* confer an increased risk for diabetes in the French Caucasian population (23).

In 2014, Wang et al. confirmed a linkage between rs1884613 of *HNF4A* gene and prediabetes risk (24).

Taken together, none of these observed associations were significant. Therefore, in the present study, these two common variants (rs1884613 and rs1884614) cannot be recognized as the T2DM and related complications susceptible risk factors in a group of Iranian population. Comparing different results of this study with the other similar studies showed that the association of *HNF4A* gene variants with T2DM risk can be affected by ethnicity background and sample size.

To investigate the precise risk or protective influence of these polymorphisms, prospective cohorts with larger sample sizes are required to be carried out in multiple settings. It is noteworthy that in the near future personalized/precision medicine of diabetes may play an important role to provide a better understanding of diagnosis, treatment and even prevention of T2DM complications according to the genetic contextin each T2DM patient.

## References

[1] Fowler MJ (2008). Microvascular and macrovascular complications of diabetes. Clin Diabetes.

[2] Whiting DR, Guariguata L, Weil C (2011). IDF diabetes atlas: global estimates of the prevalence of diabetes for 2011 and 2030. Diabetes Res Clin Pract.

[3] DeFronzo RA, Ferrannini E, Groop L (2015). Type 2 diabetes mellitus. Nat Rev Dis Primers.

[4] Fajans SS, Bell GI, Polonsky KS (2001). Molecular mechanisms and clinical pathophysiology of maturity-onset diabetes of the young. N Engl J Med.

[5] Miquerol L, Lopez S, Cartier N (1994). Expression of the L-type pyruvate kinase gene and the hepatocyte nuclear factor 4 transcription factor in exocrine and endocrine pancreas. J Biol Chem.

[6] Gupta RK, Vatamaniuk MZ, Lee CS (2005). The MODY1 gene HNF-4alpha regulates selected genes involved in insulin secretion. J Clin Invest.

[7] Argyrokastritis A, Kamakari S, Kapsetaki M (1997). Human hepatocyte nuclear factor-4 (hHNF-4) gene maps to 20q12-q131 between PLCG1 and D20S17. Hum Genet.

[8] Boj SF, Parrizas M, Maestro MA (2001). A transcription factor regulatory circuit in differentiated pancreatic cells. Proc Natl Acad Sci U S A.

[9] Hansen SK, Parrizas M, Jensen ML (2002). Genetic evidence That HNF- 1alpha- dependent transcriptional control of HNF-4alpha is essential for human pancreatic beta cell function. J Clin Invest.

[10] Thomas H, Jaschkowitz K, Bulman M (2001). A distant upstream promoter of the HNF-4alpha gene connects the transcription factors involved in maturity-onset diabetes of the young. Hum Mol Genet.

[11] Cho YS, Chen CH, Hu C (2011). Meta-analysis of genome-wide association studies identifies eight new loci for type 2 diabetes in east Asians. Nat Genet.

[12] Johansson S, Raeder H, Eide SA (2007). Studies in 3,523 Norwegians and meta-analysis in 11,571 subjects indicate that variants in the hepatocyte nuclear factor 4 alpha (HNF4A) P2 region are associated with type 2 diabetes in Scandinavians. Diabetes.

[13] Hara K, Horikoshi M, Kitazato H (2006). Hepatocyte nuclear factor-4alpha P2 promoter haplotypes are associated with type 2 diabetes in the Japanese population. Diabetes.

[14] Marathe PH, Gao HX, Close KL (2017). American Diabetes Association Standards of Medical Care in Diabetes 2017. J Diabetes.

[15] Miller SA, Dykes DD, Polesky HF (1988). A simple salting out procedure for extracting DNA from human nucleated cells. Nucleic Acids Res.

[16] Hansen SK, Rose CS, Glumer C (2005). Variation near the hepatocyte nuclear factor (HNF)-4alpha gene associates with type 2 diabetes in the Danish population. Diabetologia.

[17] Love-Gregory LD, Wasson J, Ma J (2004). A common polymorphism in the upstream promoter region of the hepatocyte nuclear factor-4 alpha gene on chromosome 20q is associated with type 2 diabetes and appears to contribute to the evidence for linkage in an ashkenazi jewish population. Diabetes.

[18] Barroso I, Luan J, Wheeler E (2008). Population-specific risk of type 2 diabetes conferred by HNF4A P2 promoter variants: a lesson for replication studies. Diabetes.

[19] Neuman RJ, Wasson J, Atzmon G (2010). Gene-gene interactions lead to higher risk for development of type 2 diabetes in an Ashkenazi Jewish population. PLoS One.

[20] Winckler W, Graham RR, de Bakker PI (2005). Association testing of variants in the hepatocyte nuclear factor 4alpha gene with risk of type 2 diabetes in 7,883 people. Diabetes.

[21] Tokunaga A, Horikawa Y, Fukuda-Akita E (2008). A common P2 promoter polymorphism of the hepatocyte nuclear factor-4alpha gene is associated with insulin secretion in non-obese Japanese with type 2 diabetes. Endocr J.

[22] Chen Z, Zhang D, Liu Y (2010). Variants in hepatocyte nuclear factor 4alpha gene promoter region and type 2 diabetes risk in Chinese. Exp Biol Med (Maywood).

[23] Vaxillaire M, Dina C, Lobbens S (2005). Effect of common polymorphisms in the HNF4alpha promoter on susceptibility to type 2 diabetes in the French Caucasian population. Diabetologia.

[24] Wang C, Chen S, Zhang T (2014). Prediabetes is associated with HNF-4 alpha P2 promoter polymorphism rs1884613: a case-control study in Han Chinese population and an updated meta-analysis. Dis Markers.

